# Congenital complete heart block in a 71-year old

**DOI:** 10.3402/jchimp.v2i4.20069

**Published:** 2013-01-07

**Authors:** Kareem Abed, Marc Mugmon, Majd Alfreijat, Robert P. Ferguson

**Affiliations:** Department of Medicine, Medstar Union Memorial Hospital, Baltimore, MD, USA

## Abstract

A case of a 71-year-old male with congenital complete heart block is discussed. Patient remained asymptomatic with stable electrocardiograms.

A 71-year old African American male presented to the emergency department with a one-day history of right upper quadrant pain suggestive of cholecystitis and was found to have complete heart block during his pre-op for possible cholecystectomy. He denied chest pain, palpitations, shortness of breath, dizziness, or syncope. Workup confirmed acute cholecystitis. Two years earlier, he had similar symptoms and was offered cholecystectomy for cholelithiasis, which he refused. Cholecystectomy was again recommended, and cardiac evaluation was performed because of a heart rate of 38.

He denied any history of birth complications, cyanosis, or other cardiac defects. He was offered pacemaker placement on multiple occasions, but refused, claiming that he was never symptomatic. Previous ECGs at age 61 (Fig. 1) and 68 revealed complete AV block, narrow QRS, and a ventricular rate of 38.

**Fig. 1:**
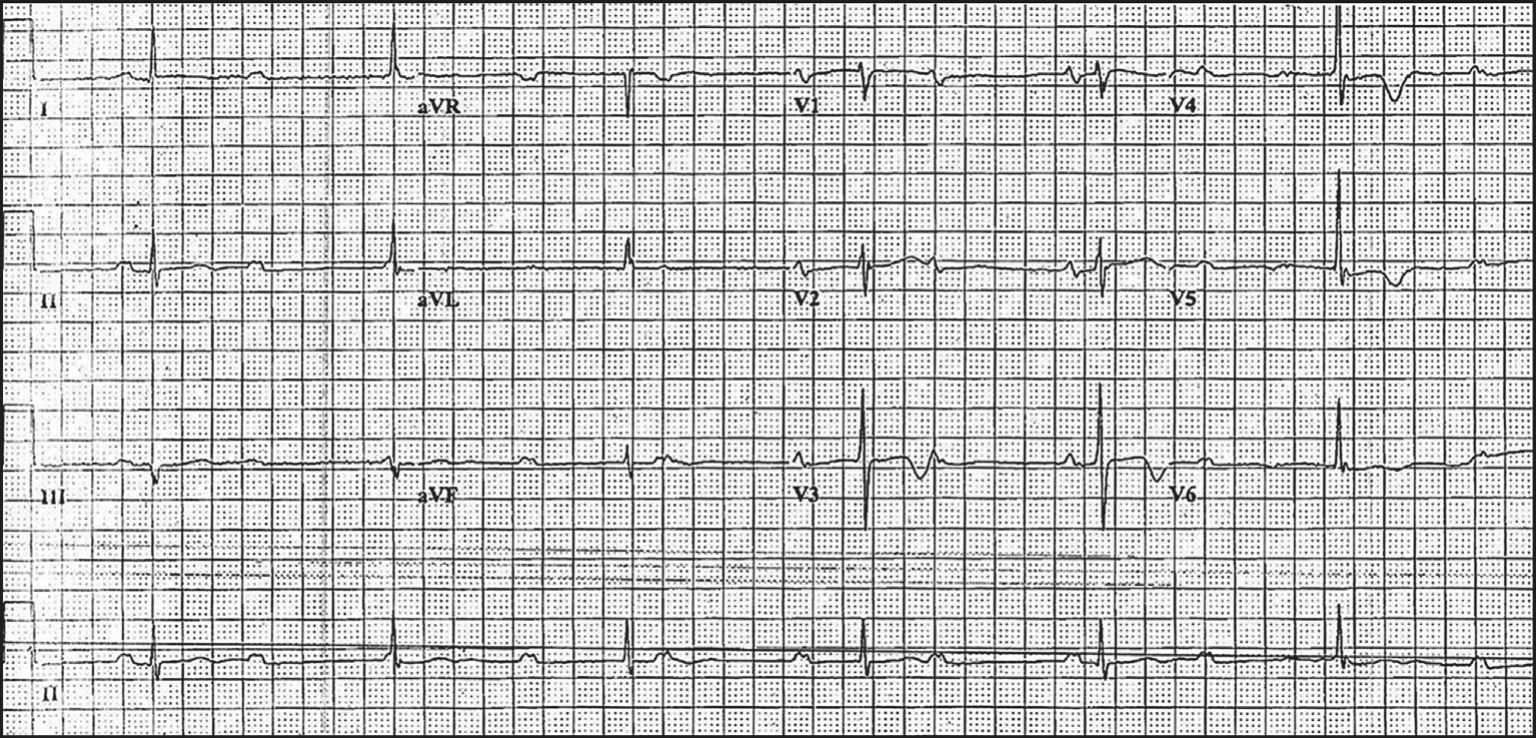
Electrocardiogram at age 61 demonstrating sinus rhythm, atrial rate 60–70, with complete AV block and narrow QRS, ventricular rate 38. Note the intra-atrial conduction abnormality.

The patient had a history of gout, esophageal reflux, multiple episodes of deep venous thrombosis, pulmonary emboli, and polycythemia. Family history was positive for lupus in his sister and there was no known history of familial heart disease. He was unaware of any maternal symptoms of lupus, and denied congenital anomalies. He stated that he had been athletic early in his life.

A physical exam revealed a blood pressure of 177/78, pulse 38, respiratory rate 18, and an oxygen saturation of 99% on room air. With the exception of bradycardia and intermittent cannon ‘a’ waves in the neck, the remainder of the exam was unremarkable. Laboratory studies revealed a hemoglobin/hematocrit at 17.3/51.1, unchanged since checked 3 years before. ANA, dsDNA, and Anti-Ro/La were negative.

Admission electrocardiogram (Fig. 2) revealed sinus arrhythmia, atrial rate of 90–100, complete AV block, narrow QRS, and a ventricular rate of 38. Echocardiography indicated normal systolic function with an ejection fraction of 60%, the presence of diastolic dysfunction, and mild tricuspid regurgitation with an estimated pulmonary artery systolic pressure of 33 mmHg.

**Fig. 2:**
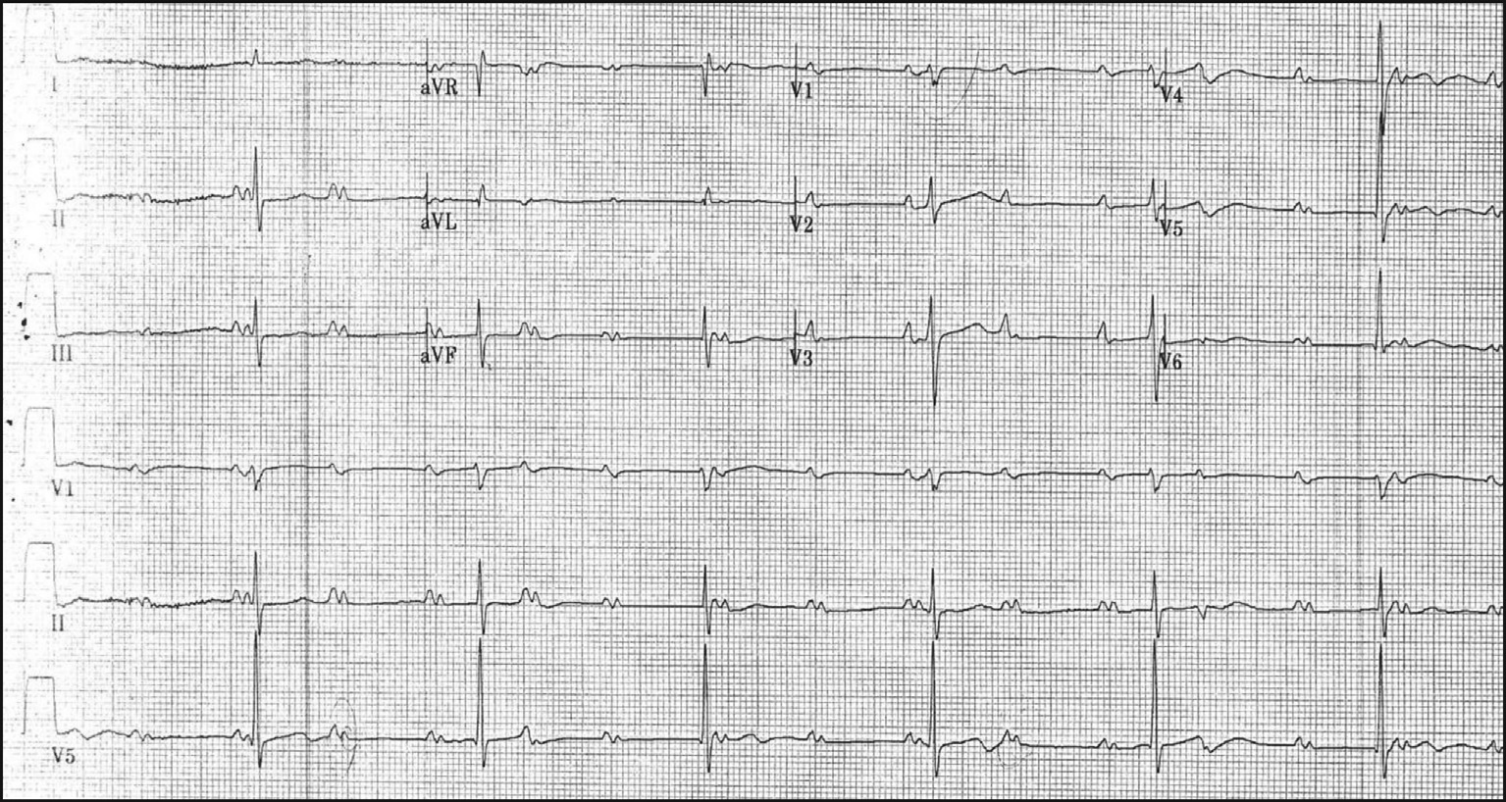
Electrocardiogram at age 71 demonstrating sinus arrhythmia, atrial rate 90–100, with complete AV block and narrow QRS, ventricular rate 38. Note the intra-atrial conduction abnormality is more prominent.

Although the patient was told he had polycythemia for the past 10 years, no etiology was found. The patient was a non-smoker, had not been exposed to second hand smoke or carbon monoxide poisoning, and denied ever being at high altitudes. Given his history of multiple deep venous thrombosis and pulmonary emboli, in the absence of endocrine disorders and pulmonary disease, polycythemia vera was considered, but was excluded with a normal erythropoietin. His absolute polycythemia was suspected as possibly being related to his heart block with an ejection fraction of 60%. According to Ikkos and Hanson, patients with congenital heart block were found to have an elevated level of total hemoglobin in response to exercise (1).

The incidence of congenital complete heart block is about 1/20,000 live births (2). It can be due to neonatal lupus in up to 90% of cases with maternal anti-La/SSB and/or anti-Ro/SSA antibodies crossing the placenta (3). These autoantibodies cause an autoimmune reaction in the AV node and may inhibit the L- and T-type cardiac calcium channels (4). Other causes include corrected transposition of the great arteries, polysplenia with AV canal defect, and other structural cardiac abnormalities. Pathologically, fibrotic changes occur in the AV node and surrounding tissues, but the rest of the myocardium is spared (4). Most cases with complete heart block of unknown etiology diagnosed after infancy remain undiagnosed for many years because the ventricular rates do tend to increase with exercise, maintaining adequate cardiac output and not leading to any symptoms.

Most deaths occur early in the neonatal period. Prenatal diagnosis increases the risk of termination, hydrops fetalis, and neonatal death. Prognosis is improved in those diagnosed in the neonatal period. Fifty percent of patients that live to adulthood develop symptoms with 10% of them dying prematurely (5). Clinical features include syncope, dizziness, shortness of breath, palpitations, and exercise intolerance. Adult patients typically report asymptomatic bradycardia, with no exercise intolerance. During exercise, patients have increased atrial and ventricular rates, with rates returning to baseline within 10 min of resting. Work capacity is conserved or slightly decreased as a result of unchanged minute volumes. Minute volumes are preserved secondary to an increased ventricular rate, stroke volume, and total hemoglobin (1).

Diagnosis is typically confirmed when an electrocardiogram is carried out due to a slow heart rate or as part of a general medical evaluation. ECG and Holter ambulatory ECG are recommended periodically after birth. A Holter ambulatory ECG is used to confirm intermittent versus constant atrioventricular block, and to determine if other arrhythmias are present. After 7 years of age, exercise testing is regularly performed. Pacemaker placement is indicated in those with symptomatic bradycardia, low cardiac output, ventricular dysfunction, wide QRS escape rhythm, complex ventricular ectopy, and in infants with a resting heart rate of less than 55 (6).
